# Nurses’ Work Concerns and Disenchantment During the COVID-19 Pandemic: Machine Learning Analysis of Web-Based Discussions

**DOI:** 10.2196/40676

**Published:** 2023-02-06

**Authors:** Haoqiang Jiang, Arturo Castellanos, Alfred Castillo, Paulo J Gomes, Juanjuan Li, Debra VanderMeer

**Affiliations:** 1 College of Informatics Northern Kentucky University Highland Heights, KY United States; 2 Mason School of Business The College of William & Mary Williamsburg, VA United States; 3 Information Systems and Business Analytics Department Florida International University Miami, FL United States; 4 Nicole Wertheim College of Nursing & Health Sciences Florida International University Miami, FL United States

**Keywords:** text mining, machine learning, blog data, COVID-19, pandemic, work concerns, stressors, natural language processing

## Abstract

**Background:**

Web-based forums provide a space for communities of interest to exchange ideas and experiences. Nurse professionals used these forums during the COVID-19 pandemic to share their experiences and concerns.

**Objective:**

The objective of this study was to examine the nurse-generated content to capture the evolution of nurses’ work concerns during the COVID-19 pandemic.

**Methods:**

We analyzed 14,060 posts related to the COVID-19 pandemic from March 2020 to April 2021. The data analysis stage included unsupervised machine learning and thematic qualitative analysis. We used an unsupervised machine learning approach, latent Dirichlet allocation, to identify salient topics in the collected posts. A human-in-the-loop analysis complemented the machine learning approach, categorizing topics into themes and subthemes. We developed insights into nurses’ evolving perspectives based on temporal changes.

**Results:**

We identified themes for biweekly periods and grouped them into 20 major themes based on the work concern inventory framework. Dominant work concerns varied throughout the study period. A detailed analysis of the patterns in how themes evolved over time enabled us to create narratives of work concerns.

**Conclusions:**

The analysis demonstrates that professional web-based forums capture nuanced details about nurses’ work concerns and workplace stressors during the COVID-19 pandemic. Monitoring and assessment of web-based discussions could provide useful data for health care organizations to understand how their primary caregivers are affected by external pressures and internal managerial decisions and design more effective responses and planning during crises.

## Introduction

### Background

The COVID-19 pandemic presented substantial challenges to health care systems overwhelmed by patients with COVID-19, creating stress on the care delivery system. For months, health professionals faced anxiety because of heavy workloads and risk of infection [1]. Overstressed nurses are now considering leaving a job that many thought to be their calling. In a survey of 6568 nurses by the American Association of Critical-Care Nurses, two-thirds of the respondents said that the coronavirus pandemic had prompted them to consider leaving the profession [2]. The ripple effects of disasters such as the pandemic may persist long after the crisis period [3].

The COVID-19 global outbreak placed health care systems under unprecedented stressors and has been a challenging time for those who work on the front lines of health care. Stress is caused by the experience of anticipating or encountering adversity in one’s goal-related efforts [4]. Facing prolonged and excessive stress may lead to nurse burnout, a state of emotional, physical, and mental exhaustion. Health providers’ experiences of burnout have been discussed in recent studies [1,5], demonstrating that it can affect not only nurses’ quality of life but also patient care. Hence, it is important to understand the work concerns and stressors behind nurse burnout during the COVID-19 pandemic.

A study on the H1N1 outbreak [6] showed that Twitter data could be used for real-time infodemiological studies, providing a source of opinions for health authorities to respond to public concerns. The availability of large volumes of user-generated content provides an opportunity for organizations to analyze the content and derive insight. In the health care context, these data can be used to capture patients’ concerns and worries or the satisfaction of staff, which can then be used to improve care [7] and working conditions. For example, user-generated content can help improve cancer care by analyzing the discussions in cancer-related communities [8] and identify mental illnesses such as depression using Instagram images taken by users during the COVID-19 pandemic [9]. Web-based content analysis is also used to determine how mask guidelines differ across nations and regions [10].

### Prior Work

There is a lack of literature on interventions for supporting health care workers during disease outbreaks. To investigate this important consideration, recent studies have used surveys to identify nurses’ stressors during the COVID-19 pandemic [11] and have shown that nursing care has been influenced by fear and isolation, making it hard to maintain the humanization of health care [12]. Survey results [11] identified 6 themes: infection-self, illness or death–others, workplace, personal protective equipment (PPE) or supplies, unknowns, and opinions or politics. Interestingly, some of the stress factors were under the control of health care organizations (HCOs). For instance, HCOs did not provide the proper equipment or put in place policies to ensure the safety and effectiveness of their staff. Many staff members felt betrayed without adequate PPE early in the pandemic and when facing furloughs and layoffs. Caught in firefighting mode, HCOs failed to sense and respond to concerns raised by clinical providers.

Questionnaire surveys can be used to investigate people’s attitudes, opinions, or knowledge related to COVID-19 [13]. However, this method limits the number of participants to a given time and location [14], which can be a constraint when addressing a global pandemic spanning an extended period [15]. Collecting and analyzing a large number of practitioner comments from social media can be an effective way to investigate and understand how the pandemic affects clinicians and their work over time. The large volume of comments across several months from different states allows us to analyze and identify topics and themes related to a complex phenomenon such as COVID-19 and its impact on health care systems longitudinally [16]. Such analysis is beneficial for health care administrators to develop effective strategies [17].

Manually analyzing large bodies of unstructured text can be resource-intensive [18]. Natural language processing (NLP), a subfield of machine learning (ML) focused on representation and interpretation of words, has been widely used to uncover useful information from large bodies of text while allowing for the validation of findings through replication [19]. Recently, NLP has been used to analyze concerns and discussions about COVID-19 from Weibo and Twitter at the early stages of the pandemic.

Although the NLP area is broad, a particular problem is the categorization of large bodies of text in an unsupervised fashion, or topic analysis. Topic modeling (TM) is an unsupervised ML application of NLP that has been used to infer patterns and group similar text or documents without needing a priori topic labels [20]. A common algorithm for TM is latent Dirichlet allocation (LDA). For example, it has been used to investigate Twitter data in real time during a natural disaster [21], analyze text comments in the health care industry [22,23], identify and understand sentiments within patient experience after total shoulder arthroplasty [24], and study health effects associated with electronic cigarette use from user-generated content from web-based forums [25].

During the COVID-19 pandemic, LDA was also used to analyze scientific publications [26] and elicit themes and emotions that measure the general public’s response to the pandemic [9,10,27]. As the pandemic evolved, further studies concentrated on symptom identification based on longitudinal data from Reddit [16] and patient web-based forums [28]. Health misinformation and conspiracies were also valuable investigation topics during this pandemic [29]. Most of these studies focused on data from patients or the general population. Fewer studies looked at the discussion and concerns from a nursing perspective. We identified 2 studies have investigated nurse burnout during the pandemic [30,31], but such studies are based on survey questionnaires that were cross-sectional in nature and only covered nurses’ willingness to care for patients in a risky environment and mental health problems at the early stages of the COVID-19 pandemic. A notable exception [32] used sentiment analysis and TM to examine posts made by nurses on social media from March 2020 to November 2020. This study focused on the detection of negative and positive emotions and sentiments of fear, frustration, exhaustion, and loneliness among nurses. Their analysis also showed how these topics changed over time.

Our study considered data drawn from nurses who served on the front lines of the pandemic. We analyzed nurses’ comments during a 14-month period to identify a comprehensive set of nursing-centric issues such as concerns about family and home impact, risk identification, work concern inventory, and nurse burnout. The data were retrieved from a web-based professional health care forum with open discussion among primarily US-based colleagues during a 14-month period to identify how work concerns evolved during the COVID-19 pandemic. To extract meaning from the forum threads, we adopted NLP techniques. We used LDA on a biweekly basis to understand temporal changes in topics.

### Study Objectives and Approach

In this study, we aimed to (1) identify themes emerging from nurse discussions related to workplace stressors during a major crisis such as a pandemic and (2) identify the salience of stressors and see the evolution of these stressors over time.

Web-based content provides an opportunity to explore and learn from events such as the COVID-19 pandemic by leveraging NLP to analyze large volumes of comments in a systematic way and investigate meaningful topics and themes. We adopted the perspective that work stressors are related to the experience of anticipating or encountering adversity in one’s goal-related efforts [4]. We guided our classification of nurses’ concerns into higher-order constructs (ie, themes) by considering the self-selected goals provided by the work concern inventory framework [33].

We complemented this framework in 2 ways. First, we adapted the framework to a health care context, where a key source of stress are interactions with patients. Concern for patients is an example of positive stress [4], the stress experienced when a person adapts positively to a challenge, and is associated with goal-oriented behavior but can also lead to role conflict, which is when the attainment of one goal hinders the fulfillment of others. Second, we considered the specific context of a pandemic in which work overload and risk management become priority concerns. The value of a goal is determined by its instrumentality in achieving other valued objectives. The goal of risk management is the preservation of the physical and human assets of the organization for the successful continuation of its operations, making self-preservation contribute to business continuity. To effectively manage pandemic-associated risks will require risk identification, risk analysis, and risk mitigation [34].

## Methods

### Overview

This study provides a year in review from the perspective of nurses working in the front lines in the United States by analyzing the data retrieved from a nurse forum that aims to unite and empower >1 million nurses across 60 nursing specialties by providing a venue for discussion (allnurses.com). AllNurses is a social media platform for nurses to share their working concerns and experiences [35] and has been previously used for data collection and analysis [36]. In this study, we collected data over a 13-month period from a specific part of the nursing forum, *COVID, Disaster, Pandemic* [37]. This part addresses “current news, experiences, and discussions on how the disaster may impact both nurses’ working and personal lives” [37].

In this forum, nurses voice concerns, share personal stories, and communicate with others regarding the COVID-19 pandemic since its outbreak, providing valuable data for investigation. We collected posts from March 2020, the onset of the pandemic in the United States, to April 2021, when the Centers for Disease Control and Prevention (CDC) eased face mask guidelines for vaccinated people and a sense of normalcy returned to health care [38]. A total of 1 year of data provided relevant insights into the perspectives of health care workers [39]. We performed a longitudinal analysis of the data to identify relevant themes related to our framework in the conversations among nurses.

### Data Analysis

**Figure 1:**
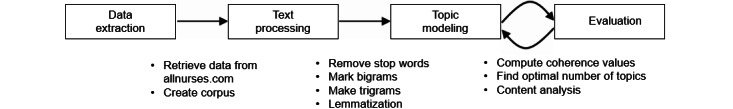
Method overview.

The data analysis stage included unsupervised ML and thematic qualitative analysis [40,41]. The unit of analysis was individual forum threads that were temporally windowed biweekly. A thread may contain posts by multiple users, and these posts are typically aligned to the name of the thread (eg, “Refusing Care of a COVID-19 Patient Due to Inappropriate PPE” posted on March 19, 2020) [42]. ML algorithms were used to segment topics and themes first, and then, following a qualitative approach, we manually reviewed a portion of the comments based on different themes to validate and explain the findings provided by the algorithms [43]. Figure 1 shows an overview of the methods used in this study.

**Figure 2:**
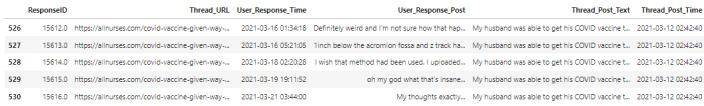
Example of forum post data.

For data extraction, a scraper engine was written using Java (Sun Microsystems) with the HtmlUnit (version 2.44.0; Gargoyle Software) package to extract data from allnurses.com for all posts within the *COVID, Disaster, Pandemic* thread. Figure 2 shows example data from the forum posts. The data set has the URL for the post, the post time, the post itself, and the thread text and thread post time. There can be many posts under the same thread, where many posts in the User_Response_Post column are part of the same thread, as shown in the Thread_Post_Text column.

We decided to perform 2 analyses: one that considered a monthly window (all posts within a month) and another that considered a biweekly window. At lower levels of granularity (ie, weekly or daily), the data set became sparse during important time frames. We created a collection of posts that served as the corpus for analysis (ie, biweekly posts).

For text processing, we then used the Natural Language Toolkit (version 3.6.5; Team NLTK) library [44] to remove stop words, split paragraphs into sentences, and then further split sentences into words (tokenize) and generate ordered combinations of words (bigrams and trigrams). Preprocessing methods play an important role in preparing data for insights and typically comprise the first step in the text-mining process [45]. The Natural Language Toolkit library includes a dictionary of common English stop words to remove (eg, *the*, *in*, *a*, and *an*) and allows for lemmatization of the corpus to reduce the dimensionality of the data set. The lemma of a word includes its base form plus inflected forms [40,45] where we considered part-of-speech nouns, adjectives, verbs, and adverbs. We also considered unigrams (eg, *nurse*), bigrams (eg, *COVID-19*), and trigrams (eg, *personal protection equipment*) as part of our feature set.

After preprocessing the data, we used TM techniques to help identify relationships among the text. We used the LDA method, which has been widely applied in NLP, social media analysis, and information retrieval [41]. LDA is an unsupervised probabilistic method that performs topic extraction by uncovering hidden structures (semantics) from a large corpus [46], where each document can be represented as a probabilistic distribution over latent topics [41]. This adds value to large document collections by discovering interpretable, low-dimensional subspaces present in the data [47]. For its implementation, we used MALLET (version 2.08), a Java-based wrapper package for Python, to perform statistical NLP, clustering, and TM [48] and Gensim (version 3.8.3; RARE Technologies Ltd) [49], an open-source Python library for TM, to build the topic models [41].

**Figure 3:**
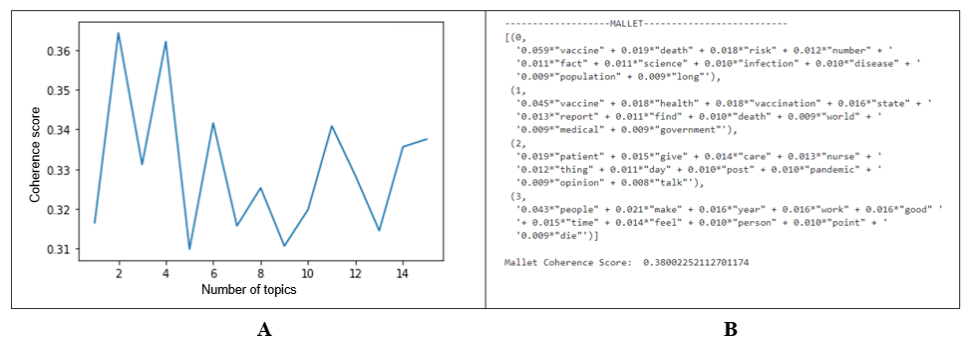
(A) Coherence score; (B) MALLET for topics.

Topic models with higher topic coherence are more interpretable (ie, words in a coherent topic have higher mutual information and, thus, are assumed to be related). Low-quality topics may be composed of highly unrelated words that cannot be fit into another topic [50] or topics that are too abstract (eg, a topic capturing the fact that nurses are discussing *COVID-19*). To select the optimal number of topics, the number of topics was bounded (n=1-16), and we chose the topic counts that gave the highest coherence score. These were further qualitatively analyzed for content, where it was found that topics too few in number tended toward being too abstract and topics too high in number tended toward having insufficient posts to produce coherent topics. Although, quantitatively, the highest performers were 2 and 4 topics, having 2 topics was found to be too abstract to be useful; therefore, having 4 topics was selected for use in the study. In Figure 3A, we present a biweekly analysis result, where having 4 topics yielded a coherence score of 0.36. Figure 3B shows an example of the 4 topics generated using MALLET. This process was repeated for biweekly and monthly levels of analysis.

TM was complemented with content analysis using an interpretive social science approach. TM removes the need for open and selective coding and theoretical sampling, enabling the analyst to condense a large corpus of narrative texts, and it becomes the analyst’s task to interpret and make sense of TM, contextualizing the topics [51].

The interpretative analysis was implemented by adapting the steps of the well-established model for thematic analysis [52]. In total, 2 researchers were designated as annotators and familiarized themselves with the data by exhaustively reading the top 10 posts in each biweekly topic generated by TM (ranked based on coherence scores). Working independently, they gave the topics theme names to ensure that the names fit the content of the posts. The researchers compared selected theme names for the labeled topics, which achieved an interannotator agreement of 68%. The 32% in conflict was due to failure to identify low-coherence themes (3%) or to interpretative disagreement, such as where one researcher favored a more abstract label (ie, *Family work balance*) whereas the other favored a more specific label (ie, *Use or abuse of leave of absence policies*). After the first round, the level of agreement reached 94%. For the remaining 6%, the underlying posts were examined together to resolve the disagreements, which left no unresolved annotations. Finally, a cluster of themes was agreed upon by the researchers, and themes were condensed into higher-order constructs; for instance, *vaccine trials* and *vaccine side-effects* were condensed into *COVID-19 vaccine*.

The grouping into higher-order themes was guided by the work concern inventory framework [33], which provides a broad view of personal self-selected goals: (1) specific job tasks, (2) coworkers, (3) supervisor or manager, (4) subordinates, (5) learning new skills or development, (6) challenge, (7) variety, (8) hours or attendance, (9) help with or feedback on tasks, (10) working conditions, (11) pay or benefits, (12) autonomy or responsibility, (13) discrimination or fairness, (14) the company, and (15) future job situation. These goals determine the type of information that individuals perceive and attend to and the individual search for feedback and should be reflected in the web-based content. We complement the issues raised by this framework by detailing the specific concerns regarding the management of pandemic-associated risks [34].

The last step in interpretative analysis involved generating higher-level narratives stitched together by themes and a sequence over time. A narrative is an account of a string of events occurring in space and time. These events do not unfold randomly but rather as an ordered series of events connected by the logic of cause and effect [53].

### Ethical Considerations

This study was conducted primarily at Florida International University, where ethics review is not needed when a study uses “anonymous samples or data available from commercial or public repositories or registries” [54]. This study used data posted publicly on an internet-based nursing interest forum. As such, no consent or ethics board review is required.

## Results

### Overview

First, we present the themes and subthemes identified by the ML system and the human-in-the-loop classification process. Then, we show the temporal evolution of each theme. Third, we delve deeper into each theme and provide a narrative of each one by analyzing the conversations over time.

### Descriptive Results

The topic analysis method identified 310 topics based on the analysis of text data from posts aggregated over 28 two-week periods from March 2020 to April 2021. The thematic analysis categorized the topics into distinct themes.

The team considered the identified topics, groups of words, and representative blog post samples in each topic; categorized the 310 topics into 58 subthemes; and then grouped these subthemes into 20 higher-order themes anchoring the theme identification in the work concern inventory [33]. For example, one topic included the unigrams *staff*, *care*, *nurse*, *nursing*, *shortage*, *number*, *find*, *provide*, *grad*, *leave*, which was categorized as a nurse shortage subtheme within the workload concern theme. Another topic included the unigrams *mask*, *room*, *wear*, *staff*, *flu*, *face*, *cough*, *time*, *pt*, *doctor*, which was categorized as mask use and PPE or risk-mitigation work concern. There were also topics with overlapping words (such as *mask*, *work*, and *home*). These were mapped into distinct themes based on the combination of words and analysis of the sentiment and meaning of the specific posts in the cluster. The final classification resulted in 20 themes, as shown in Table 1. Several topics were considered low-coherent based on the analysis of representative blog post samples (65/310, 21% of the initial topics) and were not further considered in the analysis.

Theme 1 refers to concerns about family and home impact. Nurses expressed concerns about infecting their family, leading to isolating behavior such as only talking to their children from >6 feet away with a mask. The tension with work concerns was evident, with some subthemes discussing leave of absence to resolve the tension.

A second set of themes relates to risk identification, risk-mitigation goals, and a set of work concerns related to work conditions. Themes 2 to 5 relate to risk identification: infection and fatality risk (theme 2), testing as risk identification (theme 3), virus information (theme 4), and specific nursing risk identification (theme 5). Themes 6 (PPE) and 7 (COVID-19 vaccine) refer to risk-mitigation concerns.

Themes 8 to 14 directly match items from the work concern inventory: specific job tasks, learning new skills and development, hours or attendance, working conditions, pay and benefits, autonomy and responsibility, and future job situation. These were labeled as patient care (theme 8), workload (theme 9), task completion and performance (theme 10), working conditions as safety concerns (theme 11), pay and benefits (theme 12), recognition and responsibilities (theme 13), and future job situation (theme 14).

Theme 15 refers to conversations about nurse burnout, an outcome of workplace stress. The nurses’ conversations also revealed frustrations at a broader level: frustration with misinformation, frustration with government response, and frustration with people not complying with mask recommendations. Themes 16 to 20 refer to these macrolevel concerns with potential implications in the workplace, namely, theme 16 (mitigation strategies for public health), theme 17 (mandates and restrictions), theme 18 (political, economic, and social concerns), theme 19 (government response), and theme 20 (denial and misinformation).

**Table 1:** Overview of main themes and subthemes (work concerns).

Broader theme number	Broader theme name	Subthemes^a^ (58 in total)	Summary description
1	Family and home impact	Personal choices: family vs work Family and home impact Anxiety and stress	Concern with impact on family life and work-life balance
2	Infection and fatality risk	Infection and transmission Fatality risk COVID-19 surge	Risk factors for communicable disease and concerns over mortality rate factors and rate of spread
3	Testing as risk identification	Testing policies Testing efficacy	Concerns with testing procedures, policies, and effectiveness
4	Virus information	Virus mutation science Transmission vector COVID-19 effects Information sharing	Seek to understand the pathogenic evolution of COVID-19 and transmission vectors, including communication of findings
5	Nursing risk identification	Nurse risk factors and exposure Nurse privacy Nurse self-protection Hospital regulation	Fear of being infected at work, risk factors, and exposure in the work environment
6	PPE^b^ as risk mitigation	PPE resource availability and control PPE use effects	Recommendations in the organizations regarding body protection, technology (material), management of shortage (priority allocation and reuse), and dealing with allergic and other adverse reactions to PPE use
7	COVID-19 vaccine as risk mitigation	Personal choice to get vaccinated Vaccine effectiveness Vaccine science Vaccine safety Side effects of vaccine Vaccine supply chain Optimism after vaccine Perception of vaccine risk and reward	Personal choice to get vaccinated, vaccine effectiveness, vaccine safety, side effects of vaccine, and vaccine supply chain
8	Patient care	Patient safety concerns Support system for patients with COVID-19	Concern with providing care and support to patient
9	Workload	Staff reallocation Capacity management: safety Hospital capability to respond Nurse shortage and understaffing Nurse workload and choice of floor Nurses leaving Predicting stress on health system	Nurse shortage and understaffing—anticipating demand for hospitalizations and need for adding capacity through human resources: nurse reallocation, step-up of noncritical nurses, and use of retired nurses
10	Task completion and performance	PPE Equipment Practices and procedures Vaccination and medication	Mask quality, effectiveness, and PPE use Ventilator early use and effectiveness vs other therapies and technical details on sensors and alarms Workplace COVID-19 procedures, infection prevention, intubation practices, and avoiding sample contamination Medication for patients with COVID-19 and vaccine administration information
11	Working conditions as safety concerns	Work safety concerns Supervisor or manager Learning new skills Coworkers Patient conflict	Increasing nurse-patient ratios, masks as protection in hospital, personal choices on work safety, and policies for transitioning patients Employee obligation for safety and poor management response Training for new jobs and training on critical care skills COVID-19–positive health care workers being forced to work Mask refusal: patient behavior
12	Pay and benefits	Omitted	Pay for overtime, hazard pay for frontline workers, compensation as a measure of respect, travel nurses’ compensation, and lower compensation and salary cuts
13	Recognition and responsibilities	Nurse value Nursing vs corporate interest Nurses’ union	Alignment or mismatch between nursing values and established norms and between organizational values and expectations
14	Future job situation	Future job situation	Career development and certifications
15	Nurse burnout	Nurse stress and burnout	Nurse stress and burnout
16	Mitigation strategies for public health	Omitted	Social distancing, wearing masks in public, vaccination, and herd immunity
17	Mandates and restrictions	Omitted	Lockdown scope, travel bans, need for regulations, and fairness of mask mandates and other restrictions
18	Political, economic, and social concerns	Omitted	Political polarization, prevention vs open economy, responsibility to protect the vulnerable, and costs and benefits of travel bans
19	Government response	Omitted	Role of governments regarding sanitation, misinformation, giving voice to medical experts, and impact on the economy
20	Denial and misinformation	Omitted	Web-based spread of misinformation, unscientific thinking, and conspiracy theories

^a^Not all subthemes are displayed; they were omitted for some themes for brevity.

^b^PPE: personal protective equipment.

### Temporal Analysis

#### Overview

TM allowed us to establish topics and themes and measure the prevalence of themes in each period and the change over time. The prevalence of a theme was assessed using the relative incidence rate, as shown in the following section. We built models on cross-sectional posts, namely, biweekly blog conversations, to produce high-quality topics that capture the subject within the context and period of that conversation. We then analyzed how aggregate themes evolved over time and the changes in dominant themes from period to period.

#### Themes Over Time

Table 2 shows the themes’ monthly incidence rates from March 2020 to April 2021. The rate was calculated by dividing the number of topics within a specific theme in each month by the overall number of topics identified for that month across all themes.

During a specific period, certain goals will have more influence on action than others [33], and these goals drive the information that individuals seek and the theme of web-based conversations. During the first 4 months (March 2020-June 2020), 7 themes emerged as dominant in nurses’ blog posts: concerns of identification of risk of infection and fatality risk of the new virus; nurse risk factors; work overload; lack of PPE; mitigation strategies for public health; family and home impact of the pandemic; and political, economic, and social concerns.

**Figure 4:**
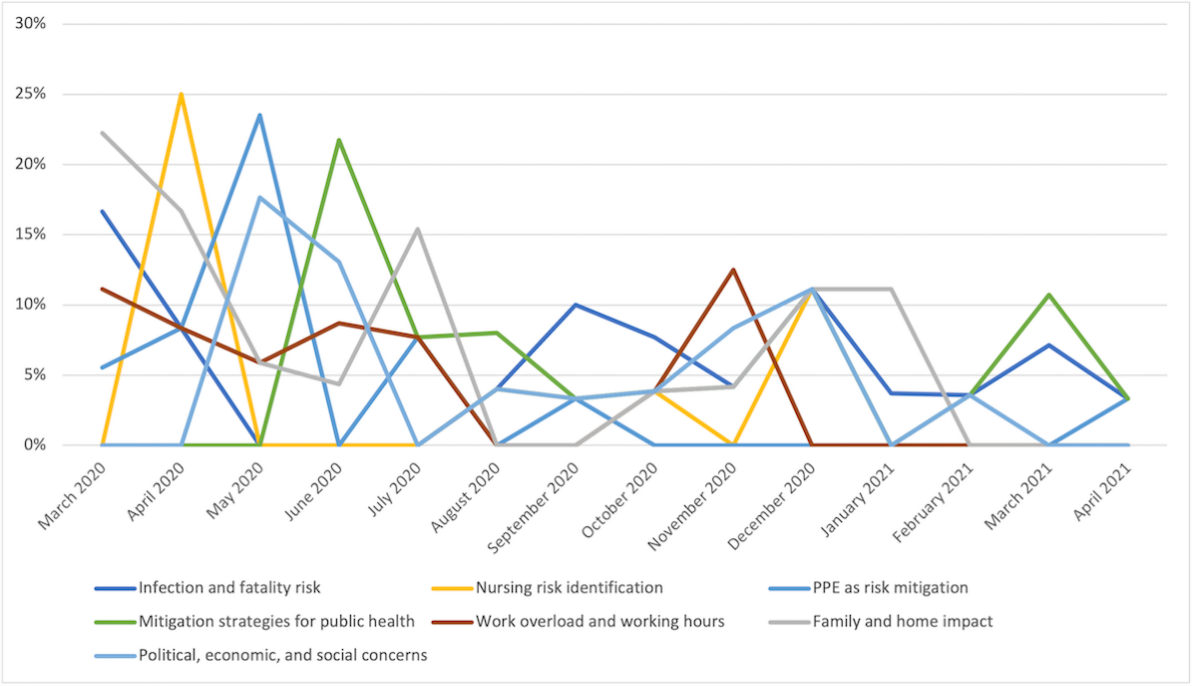
Work concerns with higher incidence rates from March 2020 to June 2020. PPE: personal protective equipment.

Figure 4 shows the evolution over time of the incidence rates of these 7 themes. The incidence of concerns with risk identification and workload decreased after the initial 2 months but increased with new waves of the COVID-19 pandemic. Concern caused by shortage of PPE peaked from April 2020 to June 2020, but this concern persisted until summer 2020, generating frustration among nurses (Figure 4).

**Figure 5:**
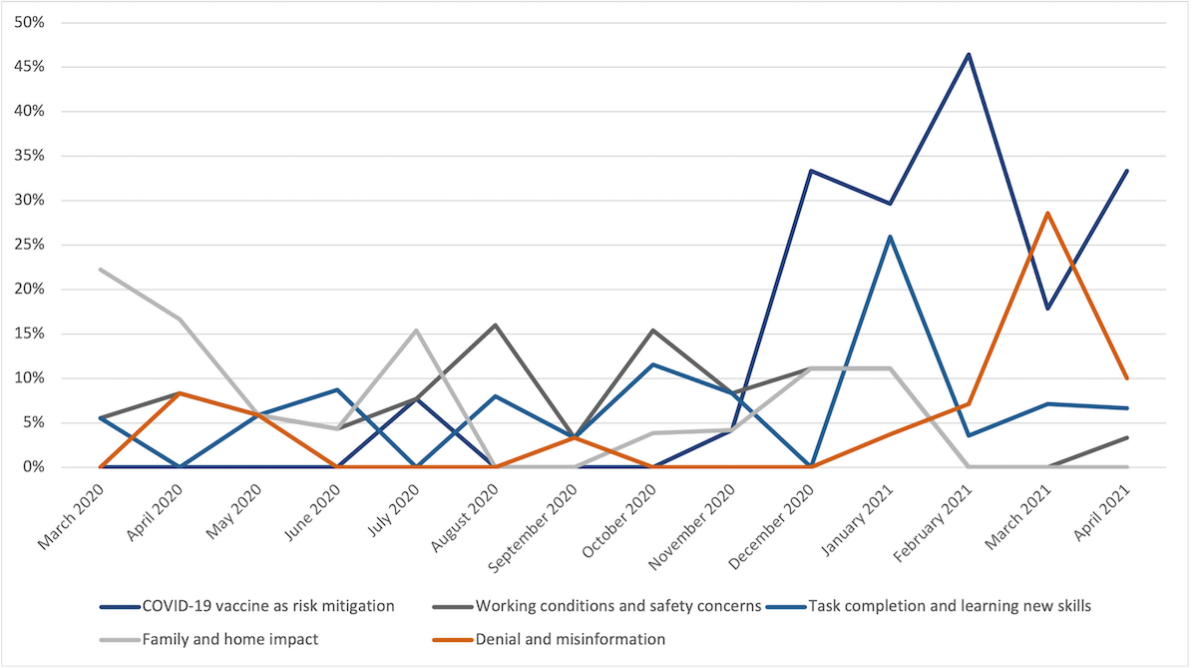
Work concerns with higher incidence rates from January 2021 to April 2021.

The work concerns during the final 4 months (January 2021-April 2021) revealed a substantially different set of concerns, as shown in Figure 5. Concerns with the COVID-19 vaccine, in particular its effectiveness as a mitigation mechanism and conflict regarding personal choice to be vaccinated, became a dominant theme. Other dominant themes included concerns about task completion (learning new skills), working conditions (safety), and denial or misinformation. Family and home impact remained a top concern.

More interestingly, the emergence of the COVID-19 vaccine was associated with a decrease in work safety concerns. Conversations on nurse risk identification and infection and fatality risk were no longer dominant; risk analysis subthemes focused on the side effects and safety of the vaccine itself, as illustrated in Figure 5 and further detailed in the following section.

**Table 2:** Temporal analysis—theme monthly incidence rate.^a^

	Year 2020											Year 2021			
	March	April	May	June	July	August	September	October	November	December	January		February	March	April
Infection and fatality risk, %	*17*	8	0	0	0	4	10	8	4	*11*	4		4	7	3
Virus information, %	6	0	0	0	0	0	0	0	4	0	0		0	7	3
Testing as risk identification, %	6	0	0	0	0	0	0	4	0	0	0		0	4	3
Nursing risk identification, %	0	*25*	0	0	0	4	3	4	0	*11*	0		0	0	0
PPE^b^ as risk mitigation, %	6	8	*24*	0	8	0	3	0	0	0	0		0	0	3
Mitigation strategies for public health, %	0	0	0	*22*	8	8	3	4	4	*11*	0		4	*11*	3
COVID-19 vaccine as risk mitigation, %	0	0	0	0	8	0	0	0	4	*33*	*30*		*46*	*18*	*33*
Work overload, %	*11*	8	6	9	8	0	0	4	*13*	0	0		0	0	0
Working conditions, %	6	8	6	4	8	*16*	3	*15*	8	*11*	*11*		0	0	3
Patient care (task completion), %	0	0	6	0	0	4	0	0	0	0	0		4	4	
Task completion and learning skills, %	6	0	6	9	0	8	3	*12*	8	0	*26*		4	7	7
Responsibilities, %	0	0	0	0	8	0	*13*	0	4	0	0		0	0	0
Pay and benefits, %	0	0	6	0	*15*	*12*	3	0	0	0	4		7	0	0
Future job situation, %	6	0	0	0	0	0	0	0	0	0	0		4	0	0
Family and home impact, %	*22*	*17*	6	4	*15*	0	0	4	4	*11*	*11*		0	0	0
Nurse burnout, %	0	0	0	0	0	4	0	0	8	0	4		0	0	0
PES^c^ concerns, %	0	0	*18*	*13*	0	4	3	4	8	*11*	0		4	0	0
Mandates and restrictions, %	0	0	6	9	*15*	0	0	4	4	0	0		0	0	10
Government response, %	6	8	0	0	0	0	0	0	4	0	0		4	0	3
Denial and misinformation, %	0	8	6	0	0	0	3	0	0	0	4		7	*29*	10
Total number of themes per month, N	18	12	17	23	13	25	30	26	24	9	27		28	28	30

^a^Italics represent an incidence rate of >10%.

^b^PPE: personal protective equipment.

^c^PES: political, economic, and social.

#### Evolution of Themes From the Work Concern Inventory

**Figure 6:**
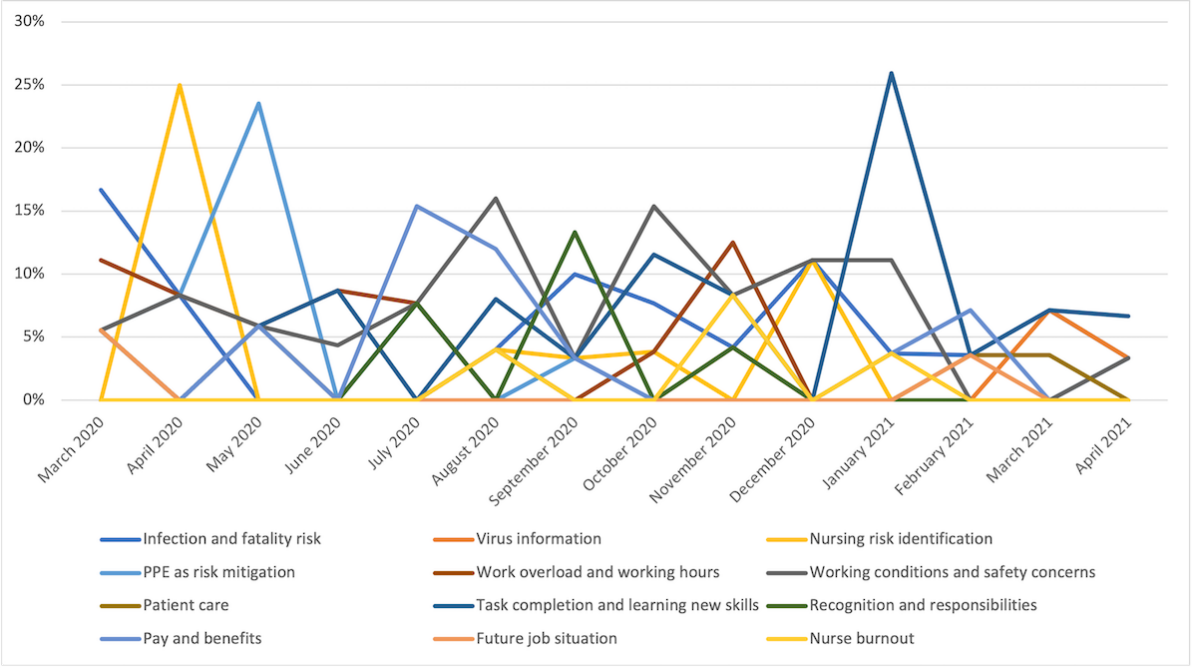
Evolution of work concerns. PPE: personal protective equipment.

Figure 6 shows the evolution of the incidence rates only of themes related to the work concern inventory. We do not display macrolevel concerns (themes 16-20), family and home impact (theme 1), testing (theme 3), or COVID-19 vaccine (theme 8). Some trends become apparent from this figure. Concerns with workload peak with the beginning of COVID-19 waves of infection in March 2020, June 2020, and November 2020. Risk identification and risk-mitigation concerns were also dominant themes in the early period and resurged with the COVID-19 waves.

Concerns regarding working conditions (safety) became dominant in the intermediate period (July 2020-November 2020) and spanned a variety of subthemes, as shown in Table 1. For instance, conversations about work conditions revealed concerns about coworkers going to work when ill. Research shows that people tend to respond differently in situations of uncertainty and fear [3], one such response being an increase in presenteeism—attending work when ill—as people either feel forced to attend because of heavy job demands (high workload and understaffing) or feel commitment to their organization and engagement with their work.

Work concerns with nurse recognition and responsibilities also became dominant in the intermediate period (July 2020-November 2020), signaling nurse frustration with managerial practices, in particular pay and benefits.

At the beginning of December 2021, as the COVID-19 vaccine became available to health care workers, perceptions of risks and work safety concerns started to fade. The sharing of practices among the nursing community for goals related to task completion and performance became a dominant theme.

Concerns with task completion involving new skills first manifested in the data in March 2020 with the beginning of the pandemic (as shown in Table 1) but only became a dominant theme 9 months later. Arguably, it takes time to create new knowledge and ensure the effectiveness of a new practice to feel confident sharing on social media. However, the focus of attention on concerns regarding risk identification, analysis, and mitigation during a specific period may have enabled those personal goals to have more influence on action than others in determining the information that nurses seek.

### COVID-19 Narratives

#### Overview

A narrative is an account of a sequence of events connected by the logic of cause and effect. Narratives are the primary way in which we understand and give meaning to experiences and can form the basis for theoretical explanations of organizational phenomena [55]. TM based on biweekly blog conversations produced topics that captured the subject within the context and period of that conversation. Further analysis of the prevalence and change in topics within each theme allowed us to summarize sequential patterns of events, providing an understanding of COVID-19 narratives that would otherwise be difficult to identify.

There are five key properties that provide structure to narratives [53]: (1) a sequence in time, (2) focal actors, (3) an identifiable voice, (4) standards to judge the actions of the actors, and (5) context. Our study began with the onset of the pandemic in March 2020 and ended in April 2021 with the easing of CDC guidelines, providing a sequence for the narratives.

As we made sense of patterns of events during this period, we considered the other 4 features of a narrative structure: the focal actors were the nurse professionals, whose comments reflected their roles and self-selected goals; nurses also provided a specific point of view of the narratives (identifiable voice); the organizational culture (including values and belief systems) of US health care delivery systems provided the standards for judging actions; and the interpretation of the events was tied to a specific context, the COVID-19 pandemic, which gave meaning to the narratives.

Next, we highlight 3 narratives from our study, sustaining an explanation of changes in work concerns and the connection between events and their consequences, namely, nurse frustration and burnout.

#### Managing PPE: a Case for Social Listening

The 2 subthemes under PPE as risk mitigation are “PPE resource availability and control” and “PPE usage effects.” A detailed analysis of the evolution of these themes over time, as reported by nurses on the forum, created a meaningful narrative.

There was a widespread sense that HCOs did not provide the proper equipment or put in place policies to ensure the safety and effectiveness of their staff. Our results demonstrate first the concern with exposure to patients and lack of availability of PPE (March 2020):

This is a part of my resignation letter. With the current issues surrounding the Covid 19 crisis it has become clear that the hospital is not prepared with adequate supplies to keep us all as safe against contracting the virus. We were told by management that we do not have enough N95 masks and have to conserve them. We are asked to reuse N95 mask and put it in a paper bag for a week of work with patients on Covid 19 section of the unit. The same applies to the gowns and face shield: one gown has to last for all assigned patients for a week. We have no shoes cover and hair cover that will allow the virus to spread around the unit.

Caught in firefighting mode relative to PPE and other equipment, HCOs forged ad hoc responses that added further instability, as explained by the following post:

We are also told to use surgical masks for a week for all other assign patients that are not suspected to carry Covid19 virus. This contradicts everything we have been taught previously from Infectious Control and the CDC. Even now the policies in the hospital are changing daily for more unsafe that brings chaos, confusion, and anxiety among the staff.

As time evolved, the topics on the PPE theme revealed further concerns with the purpose of wearing masks, shortage, and complaints about work-arounds from HCOs, leading to user innovation in mask development and discussion of protection options and effectiveness (March 2020 to May 2020), which shifted to concern regarding the consequences of wearing PPE, allergies, and solutions (August 2020) and recommendations and sharing of information on specific task completion practices (August 2020).

By failing to listen, organizations did not adapt to coping problem-solving behaviors of clinicians and envisioned work-around solutions that fell short of meeting standards, contributing to a feeling of high risk of exposure, unsafe work environments, and low valorization of staff:

Knowing that inconsistent hospital policy regarding reuse of PPE will fail to keep me safe from contracting the virus I obtained a half face respirator on my own and was forbidden to use it by the nursing supervisor. It a scientific fact that P-100 mask provides better protection against a variety of foreign respiratory particles than N95 and yet I was told by the supervisor that it is against hospital policy now and must be approved by infection control. The hospital should have already UV lights to disinfect our PPE if we have to reuse it and the rooms after Covid patients. I can see that important decisions were not made in a timely matter and action plan not implemented right away to prepare our hospital especially when we were warned by what was happening in New York City more than 2 weeks ago. Hospital had time to prepare but waited till last minute and what it did it locked up and rationed PPE. In this unfortunate situation even asbestos workers with their PPE gear are better equipped to work with Covid19 than us healthcare workers in our PPE given by hospital.

Further involvement of nurses in the design of safety practices could have contributed to effective solutions while promoting nurse well-being [56]. The role conflict created by the lack of PPE became a work stressor that was associated with nurse resignation:

These are the reasons why I am submitting my resignation effective immediately. I am perfectly willing to work and continue help to care for patients conflicted with this virus but with proper PPE. On the other hand, I cannot be asked by my employer to work in the environment that puts my life in danger.

#### A Narrative of Nurse Disenchantment

A set of themes shared unigrams in the ML grouping, such as “nurse,” “risk,” and “pay,” and human-in-the-loop thematic analysis often found the post content entangled, such as hazard pay to cover nurse risk factors or compensation as a measure of respect for nurse worth. We show in Table 3 the evolution of conversations regarding nursing risk identification (theme 6), pay and benefits (theme 12), recognition and responsibilities (theme 13), future job situation (theme 14), and nurse burnout (theme 15).

Pay and benefits became a dominant subtheme in July 2020 to August 2020, representing 60% (3/5) of the topics. The topics within pay and benefits were diverse, including pay rates for nurses under different conditions and pay for overtime. Conversations regarding compensation also addressed the fairness of hazard pay for frontline workers and the high pay rate associated with travel nurses’ compensation. Nurses struggled with unpaid leave for those who tested positive for COVID-19, salary cuts, and furloughs.

In July 2020, we saw the first concerns regarding nurse recognition and the emergence of nurse burnout as a new subtheme. Nurse recognition emerged as a dominant theme, and the topics within compensation reflected the change of tone in the conversation; compensation emerged as a measure of respect for nurse responsibility and worth and was deemed less important than enabling a reduced workload to prevent exhaustion:

Found out our surgical ICU unit (which houses critical covid patients) and ER got a few $$/hour increase for covid hazard pay. Meanwhile my unit which is the primary covid telemetry/med-surge unit was denied. Administration and upper management has been honoring their initial plan of keeping us at 3:1 as long as staffing allows which it has been. If I had to choose I would definitely choose 3:1 ratio over $ increase...It’s crazy how much time you save by having one less patient.

Burnout is characterized by emotional exhaustion, cynicism, detachment, and a sense of lack of accomplishment [57]. The following nurse quote illustrates the feeling of “moral distress”—not being able to do their job and care for patients the way they believed they should:

The nurse’s career and duty are to use good judgment in treating patients—not to be a martyr especially those who have dependent children. The employer’s job is to enforce rules that protect nurses other staff and other patients.

Both the policy of unpaid sick leave and the practice of isolation from family for fear of infection created frustration and negative well-being among nurses.

**Table 3:** Temporal analysis—nursing risks, pay and benefits, and recognition.^a^

	Year 2020, %											Year 2021, %			
	March	April	May	June	July	August	September	October	November	December	January		February	March	April
Nurse risk identification	0	1	0	0	0	1	0	1	0	1	0		0	0	0
Pay and benefits	0	0	1	0	2	3	1	0	0	0	1		2	0	0
Future job situation	1	0	0	0	0	0	0	0	0	0	0		1	0	0
Nurse recognition	0	0	0	0	1	0	4	0	1	0	0		0	0	0
Nurse burnout	0	0	0	0	0	1	0	0	2	0	1		0	0	0

^a^The table reports the number of topics in each month categorized under the subtheme.

#### The COVID-19 Vaccine Narrative

The data support the idea that the vaccine was an effective risk-mitigation mechanism [58], producing a substantial shift in the work concerns of nurse professionals. Table 4 shows the sequence of conversations about the COVID-19 vaccine. Although the initial focus in December 2020 and January 2021 was on vaccine safety and assessment of side effects, the dominant theme shifted in February 2021 to the right of refusal and matters of personal choice in receiving the COVID-19 vaccine.

The initial concerns (March 2020 to June 2020) were spread among nurse risk factors, continuation of nurse training, and compensation. The posts related to fear of being infected peaked in April 2020, followed by a decrease in the rate of posts and another increase during the summer and in December 2020 with surges in COVID-19 infection across the United States.

There were early concerns regarding the science behind the COVID-19 vaccine (July 2020), but the COVID-19 vaccine only became a dominant theme in January 2021. A total of 4 main subthemes drove the vaccine narrative: concerns with effectiveness as a risk-mitigation mechanism; vaccine safety and concern with side effects; and, finally, individual willingness to receive the vaccine.

First, there was a sequence of conversations regarding the effectiveness of the vaccine as a mitigation mechanism. The comments on this topic focused on whether the COVID-19 vaccine was effective, supported by a discussion of the science behind it. Some posts suggested that patients may have fewer symptoms after vaccination based on previous experience with the influenza vaccine:

Even when people get the flu after receiving an influenza shot that they tend to get less severe forms of the flu and to spend less time in the hospital.

Others commented that the COVID-19 vaccine should work similarly to previous vaccines:

We have proven that previous vaccines work for other viruses that have infected us! If you don’t believe in the RNA vaccine then take the conventional vaccine.

However, many nurses doubted the effectiveness of the COVID-19 vaccine at the early stage of vaccine implementation (from December 2020 to February 2021), arguing that the effectiveness rate was low and that the sample size of experiments was not enough at the early stage of COVID-19 vaccine development.

**Table 4:** Temporal analysis—COVID-19 vaccine subthemes.^a^

	Year 2020, %											Year 2021, %			
	March	April	May	June	July	August	September	October	November	December	January		February	March	April
Vaccines: personal choice	0	0	0	0	0	0	0	0	0	0	1		5	0	5
Vaccine effectiveness	0	0	0	0	0	0	0	0	0	1	1		3	2	2
Vaccine science	0	0	0	0	1	0	0	0	1	2	0		2	1	0
Vaccine safety	0	0	0	0	0	0	0	0	0	0	2		2	1	2
Side effects of vaccine	0	0	0	0	0	0	0	0	0	0	3		0	0	1
Optimism after vaccine	0	0	0	0	0	0	0	0	0	0	0		1	0	0
Perception of risk and reward in vaccine	0	0	0	0	0	0	0	0	0	0	0		0	1	0
Vaccine supply chain	0	0	0	0	0	0	0	0	0	0	1		0	0	0

^a^Reports number of topics in each month categorized under the subtheme.

After more people were vaccinated and data from the CDC showed vaccine effectiveness, the narrative changed to focus on the quality of the COVID-19 vaccines. People compared COVID-19 vaccines from Pfizer, Moderna, and Johnson & Johnson. These posts emerged in February 2021:

The efficacy rates of the Pfizer and Moderna vaccines are just much more promising to me than J&J. Although the flu vaccine is typically only around 40-45% effective each year and J&J’s COVID vaccine outperforms those statistics.

Vaccine safety emerged as the second concern. Some nurses believed that the COVID-19 vaccine was safe based on scientific knowledge of messenger RNA:

mRNA technology is new but not unknown. They have been studied for more than a decade. mRNA vaccines do not contain a live virus and do not carry a risk of causing disease in the vaccinated person. mRNA from the vaccine never enters the nucleus of the cell and does not affect or interact with a person‚ DNA.

However, others worried about COVID-19 vaccine safety as it was still in the experimental stage, and data from the CDC showed that the COVID-19 vaccine was not 100% safe, with a certain death rate. Beyond the concern about the early stage of COVID-19 vaccine development, some individuals did not trust any type of vaccine (February 2021 to March 2021):

It also stated that long term side effects were unknown due to still being in clinical trials that it may or may not protect against covid-19 and that if injury occurs due to the vaccine it is NOT covered under the vaccine injury program since it is NOT FDA APPROVED. After reading and rereading the paperwork carefully I chose not to get the vaccine.

Don’t feel I should be forced to take one either. It’s my choice. Medical facilities are loaded with diseases and contagions you can pick up at any time and proper precautions were always in place to limit exposure. I am not one to have myself injected with a virus...alive or dead when I am in good health either and take good care of myself.

The third concern was the side effects of the vaccine. Several nurses shared the care experience of Guillain-Barré syndrome from the influenza vaccine and worried that it may also be observed after COVID-19 vaccine injection:

I can only say what I’ve seen re GB and the flu vaccine. I took care of 3 patients they were all over 50. One man was totally paralyzed but could breathe on his own. I was told he eventually recovered but it took a year, and he was so despondent that he killed himself. I remember him clearly 75-year-old active healthy guy who rode his motorcycle across country and then felled by the flu vaccine. It was so sad and from that time I avoided the flu vaccine till it was made mandatory.

Others believed that the COVID-19 vaccine had severe side effects based on scientific articles:

Local reactions to the vaccine were generally mild. About half the participants receiving mRNA-1273 experienced moderate to severe side effects‚ such as fatigue muscle aches joint pain and headache‚ after the second dose. In most volunteers these resolved within two days. One potential concern about COVID-19 vaccines is an unusual phenomenon called vaccine-associated enhanced respiratory disease or VAERD.

In addition, nurses shared their own experiences with the side effects of the COVID-19 vaccine. Some people had mild side effects, whereas others had severe side effects:

Sore arm after 1st dose for about 2 days otherwise no side effects. Got 2nd dose two weeks later. Started feeling crummy about 16 hours out- sore muscles/joints headache nausea general malaise. Lasted less than a day and then felt completely fine.

Some really got hit hard after the first injection others it was the second. Same with our patients the more fragile and older residents spiked some pretty high fevers and GI upset but with Tylenol hydration and plenty of rest they recovered quickly.

I got my 1st dose Moderna yesterday. I’m sicker than I ever was with actual covid I had a very mild case. Today I have a high fever muscle aches dizziness and a pounding headache.

The fourth concern related to the personal choice of being vaccinated. Some people believed that the COVID-19 vaccine would be mandatory in most places and would be related to personal social benefits such as a passport, taxes, schools, and jobs:

I believe (in the next 1 to 5 years) failing to have the Covid vaccine may involve giving up things like being able to have a passport attend certain sports or concert venues or even to visit certain private companies or federal facilities as well as exclude you from many institutions of higher learning and many job opportunities. Ultimately you may have to ask yourself how much am I willing to give up? However not getting one would have meant no tax deductions no passport exclusion from most scholarships (or even applications to universities he was homeschooled so school until college wasn’t and issue) not to mention exclusion from 95% of all jobs and social security benefits.

However, others believed that a COVID-19 vaccine should not be mandatory. They indicated that the vaccine should be optional and expressed distaste for strong-arm tactics to force participation, such as fear of job and other social benefit loss, citing that the safety and side effects of the vaccine had not been fully examined with a high standard yet:

If you start using well you could choose to take the vaccine then it is an almost endless list. When it comes to something like a vaccine that has both risks, benefits, and involves a medical intervention with regard to your immune system it should always be a choice without fear of job loss or other issues.

I don’t believe the vaccines should be mandatory at this time due to concerns about both the autonomy of healthcare personnel the lowered bar for safety that comes with emergency use authorization rather than full FDA approval and the possibility of stirring up a bigger backlash against the vaccine.

The argument of mandatory vaccination seemed to transcend the science and policy perspectives into that of morality. Several posts indicated that nonimmune people might transmit COVID-19 to other people, particularly vulnerable populations such as pregnant women, older adults, and children. Being more vulnerable, they may develop severe symptoms. They believed that social morals should be one of the major considerations when making the decision of receiving the COVID-19 vaccine:

You go to the grocery store and unknown to you infect others. A pregnant woman gets ill and dies taking her baby with her. Her children at home are now motherless and the husband is heartbroken and begins drinking neglecting his parenting.

The COVID-19 vaccine narrative also contributes to understanding the phenomenon of nurse disenchantment and offers insights for organizational action regarding mandatory vaccination and personal choices. Although workplace safety is a priority goal, safety practices that undermine employee satisfaction can lead employees to resent management, avoid strict adherence to policies, and resist whether overtly (ie, quitting) or covertly (ie, calling out) [3]. Nurses’ viewpoints on coworkers’ choices regarding vaccination and management’s stance on vaccine mandates present an opportunity for crafting informed organizational responses.

## Discussion

### Principal Findings

This study used posts from a professional nurse web-based discussion platform to identify nurses’ work concerns during the COVID-19 pandemic.

The data analysis revealed the emergence of work concerns related to risk identification and mitigation, that is, reducing exposure to the risk and the likelihood that the risk will occur. These concerns revealed personal goals in these domains that became critical determinants of behavior in organizational settings [33]. Nurses still expressed concerns about task completion and operating with high performance. In fact, the theme became dominant in later stages of the timeline as risk-mitigation mechanisms came in full force, namely, the vaccine.

Our analysis also shows that the government’s inconsistent posture in implementing policies contributed to the emergence of concerns among nurses, a finding consistent with previous studies on public sentiment during the COVID-19 pandemic [59].

Managerial practices have unintended consequences and may create trade-offs that amplify the stress related to work concerns. For instance, job rotation serves to make work more interesting by providing variety but can enhance stress [56]. Regarding compensation practices, employees are typically more motivated and satisfied when they receive the rewards that they feel they deserve [60]. However, incentive compensation practices can undermine employee well-being when they introduce inequity into the organization, which is a fairness work concern. The nurse narratives revealed concerns with nurse pay cuts and furloughs in some areas as well as concerns over the merit of hazard pay and the high compensation for travel nurses. These practices were intended for the dynamic adjustment of hospital capacity to meet demand but seemed to leave nurses angry as they deemed them unfair.

We differentiate from previous studies that have analyzed data from social media outlets such as Facebook and Twitter [32] by focusing on a domain-specific outlet, a professional nursing forum. Our work also used ML to categorize the evolution of nursing conversations during the pandemic. We then used a theoretical framework to filter work concerns. Our results show that the conversations were more focused and job-related and less personal.

In our study, we used text created by nurses to infer work-related concerns that affect employee satisfaction and, eventually, their productivity and organizational performance. We contributed to a stream of studies that use text on social media to infer organizational performance [9]. In addition, our study identified new discussion topics regarding COVID-19 work concerns when compared with previous studies [9,32]. Finally, adopting a temporal analysis, our study explored how conversations evolved within themes and offered a narrative of work concerns.

### Limitations

There were some limitations to this study. The main limitation of our study lies in the reliance on blog data primarily from a single country, reducing the possible generalization of results to other populations. Another limitation is that the data collected from the web-based media reflect perceived work context characteristics, which could exhibit personal bias and, consequently, not be a true representation of the work environment. Although narratives require an identifiable voice—in our case, nurse professionals—future studies may consider multiple points of view, including other stakeholders such as physicians and administrators. In addition, this study did not conduct sentiment and emotion analyses of comments. Future research can explore public emotions by analyzing comments written in response to original posts.

### Practical Implications

The results of this study may assist health care managers and policy makers in being active observers of professional participation and activity in web-based media. Social listening is an important way to gauge health care professionals’ concerns and responses. When faced with unexpected events, health care leaders need to devise organizational strategies that support physicians, nurses, and care team members, ultimately promoting organizational justice, which can include manageable workloads, flexibility to facilitate family-work balance, and ensuring that clinicians feel valued and heard [61]. Several suggestions emerged from the data: leaders must communicate best practices clearly; manage expectations; clarify work hours; and provide sufficient resources, including effective PPE. Leaders should aim to monitor clinician wellness and proactively address concerns related to the safety of clinicians and their families. During the pandemic, clinicians should be encouraged to openly discuss vulnerability. Frontline clinicians may individually and collectively identify concerns that arise while facing the reality of the pandemic while considering the importance of team morale and protecting one’s emotional strength. HCOs can provide opportunities for social support during crisis situations using internal web-based forums where nurses can share the stress factors that are affecting their work, support one another, and make suggestions for workplace adaptations during a pandemic crisis.

An interesting result is that the risk mitigation offered by the COVID-19 vaccine enabled a shift in focus from concerns regarding risk identification, analysis, and mitigation to task completion and performance and sharing of new practices and skills. It is important for HCOs to sense the personal goals that become dominant work concerns during a specific period as they will have more influence on action and on the information that clinicians seek than other goals. During a pandemic, organizations may deploy mechanisms that focus on task completion and performance based on new skills earlier by seeking to address other self-selected personal goals that have become dominant.

Our findings regarding the evolution of work concerns also inform the design of managerial practices during periods of uncertainty and fear, such as a pandemic. For instance, previous studies suggest that managers achieved positive well-being synergies by involving employees in the design of safety practices [56]. Safety practices that undermine satisfaction can lead employees to resent their supervisors and avoid following the policies. A nurse discussed how mask wearing and social distancing were not followed by coworkers, creating a sense of risk of being infected at work. Some coworkers may prefer the comfort and image of being without safety equipment during breaks or when not close to patients. While responding to a pandemic, managers can collect information on employees’ attitudes toward current practices using web-based media to sense and respond to these trade-offs.

### Conclusions

Overall, this study stresses the importance of understanding the experiences of nurses during a period of uncertainty and fear created by the COVID-19 pandemic. Nurses serve on the front lines of the health care delivery system, which, in general, was not prepared to respond. Responding to this situation triggered work concerns and produced tensions. The study findings regarding the evolution and interrelation of work concerns serve as a basis for articulating the lessons learned, which will be useful to various government agencies, hospitals, organizations, and communities that wish to design managerial practices that address nurses’ work concerns more effectively.

## Abbreviations

CDC: Centers for Disease Control and Prevention
HCO: health care organization
LDA: latent Dirichlet allocation
ML: machine learning
NLP: natural language processing
PPE: personal protective equipment
TM: topic modeling
